# Toxicity mechanism analysis of cGAS-STING-TBK1 signaling pathway small molecule modulator based on network toxicology and molecular docking strategy: quinacrine acetate as an example

**DOI:** 10.3389/fchem.2025.1584588

**Published:** 2025-04-22

**Authors:** Jinchao Zhu, Qingyuan Lin, Honglin Zhu, Siqi Xie, Shengdong Nie

**Affiliations:** ^1^ School of Health Science and Engineering University of Shanghai for Science and Technology, Shanghai, China; ^2^ Department of Pathology, Shanghai Ninth People’s Hospital, Shanghai Jiao Tong University School of Medicine, Shanghai, China; ^3^ Sichuan Cancer Hospital, University of Electronic Science and Technology of China, Chengdu, China

**Keywords:** quinacrine acetate, network toxicology, molecular docking, cGAS-STING-TBK1, respiratory toxicity

## Abstract

**Objective:**

This study aims to investigate the toxicity characteristics and mechanisms of quinacrine acetate, a small molecule modulator of the cGAS-STING-TBK1 signaling pathway, and to establish and validate the application value of network toxicology analysis strategy.

**Methods:**

ProTox and ADMETlab platforms were used to evaluate the toxic effects of quinacrine acetate on human tissues and organs. Potential targets associated with quinacrine acetate toxicity were identified through ChEMBL, STITCH, GeneCards, OMIM, and TD databases. GO and KEGG analyses were employed to elucidate related functions and molecular mechanisms. STRING and Cytoscape software were utilized to identify key hub genes, while molecular docking validation was performed using the CB-Dock2 database. Based on toxicity analysis results, COPD was selected as a disease model, and GEO database was used to analyze the expression characteristics, immune correlation, and drug target value of hub genes in COPD.

**Results:**

ProTox and ADMETlab analyses revealed that quinacrine acetate exhibited significant toxicity to the respiratory system (toxicity level 4, risk coefficient 0.959). Through integrated multi-database analysis, 14 potential targets related to quinacrine acetate-induced respiratory system toxicity were identified. GO and KEGG pathway analyses indicated that quinacrine acetate-induced respiratory toxicity was primarily mediated through metabolic pathways. Network analysis via STRING and Cytoscape identified AKT1, PLA2G4A, and ALOX5 as three core targets. Molecular docking results confirmed strong binding affinity between quinacrine acetate and these core targets. In COPD patients, PLA2G4A and ALOX5 showed significantly upregulated expression, with hub gene ROC curve AUC value reaching 0.829, demonstrating good diagnostic value. Further immune correlation analysis revealed that ALOX5 and PLA2G4A were closely associated with various immune cell expressions and served as targets for multiple drugs including histamine, melittin, and formic acid.

**Conclusion:**

This study demonstrates that quinacrine acetate may influence the progression and risk of respiratory system diseases by regulating metabolic pathways. The findings provide not only a theoretical foundation for understanding the molecular mechanisms of quinacrine acetate-induced respiratory toxicity but also new perspectives and methodological references for evaluating the toxic effects of small molecule compounds in respiratory diseases. Therefore, we demonstrates the practical application value of network toxicology as an efficient predictive tool for identifying potential toxicity targets and pathways, which can guide subsequent experimental validation and provide mechanistic insights that traditional toxicology approaches might miss.

## Introduction

With the advancement of biomedical research, small molecule compounds have become increasingly important tools for modulating cellular signaling pathways in disease treatment, such as in glioma (Munson et al., 2013), sepsis (Su et al., 2023), and Parkinson’s disease (Guo et al., 2023). However, while exerting therapeutic effects, these small molecule modulators may induce toxic effects on specific tissues or organs, limiting their clinical applications (Fairman et al., 2021).

Quinacrine acetate (Qac), as a key small molecule modulator of the cGAS-STING-TBK1 signaling pathway, demonstrates significant immunostimulatory activity (Li and Chen, 2018; Xia and Konno, 2016). This compound shows great potential in enhancing anti-tumor immune responses through precise regulation of the cGAS-STING-TBK1 signaling pathway (Huang et al., 2022; Woo et al., 2014). As a critical regulatory pathway in innate immune responses, the cGAS-STING-TBK1 signaling pathway has received widespread attention in recent years, with small molecule modulators of this pathway showing potential value in treating various diseases (Wang et al., 2020; Xu et al., 2023; Yang et al., 2023). Specifically, quinacrine acetate can address the issue of poor immunogenicity in various tumors, thereby improving the overall effectiveness of cancer immunotherapy. By regulating innate immune responses, quinacrine acetate exhibits a unique mechanism of action in enhancing immune activation in the tumor microenvironment. However, as a small molecule modulator of this pathway (Ding et al., 2020), a systematic evaluation of the therapeutic effects and potential toxicity of quinacrine acetate has not been fully elucidated.

Traditional toxicological research methods, typically based on *in vivo* and *in vitro* experimental models, are time-consuming, costly, and often fail to comprehensively reveal the molecular mechanisms of toxic effects (Settivari et al., 2015). With the rapid development of bioinformatics and systems biology, network toxicology has emerged as a novel toxicological research strategy that provides new insights for comprehensively analyzing compound toxicity mechanisms by integrating multi-omics data and constructing molecular interaction networks (Zhang et al., 2023; Harrold and Ramanathan, 2013; Tao et al., 2013). Molecular docking, not only serving as computer-aided drug design (Pinzi and Rastelli, 2019; Huang, 2023), can also be used to determine the material basis of toxicants based on compound-target interactions and affinity. Furthermore, the application of molecular docking technology provides molecular-level evidence for elucidating the interactions between small molecules and target proteins (Kitchen et al., 2004).

While quinacrine acetate is primarily studied as a cGAS-STING-TBK1 pathway modulator, our research hypothesis is that its toxic effects may result from off-target interactions with proteins beyond this canonical pathway. Such off-target effects could involve cross-pathway regulation through protein conformational changes or allosteric modulation. This study aims to identify these potential off-target interactions and their downstream consequences, providing a more comprehensive understanding of quinacrine acetate’s pharmacological profile.

## Materials and methods

### Compound toxicity prediction

The chemical structure of Qac was analyzed using PubChem (Kim et al., 2021) (https://pubchem.ncbi.nlm.nih.gov/). ProTox (https://tox.charite.de/protox3/index.php?site=home) and ADMETlab (https://admetlab3.scbdd.com/server/screening) online tools were employed to predict the toxicity risk of Quinacrine acetate to different human tissues and organs based on its chemical structure. The target organ for focused investigation was determined according to the toxicity prediction results. ProTox and ADMETlab platforms were selected based on their comprehensive coverage of respiratory toxicity endpoints and validated prediction algorithms. ProTox uses a consensus approach combining 2D similarity, fragment propensities, and target binding probability, while ADMETlab integrates machine learning models trained on over 30 ADMET endpoints. The toxicity risk coefficient (0.959) represents the probability of respiratory toxicity based on structural alerts and physicochemical properties, with values >0.7 considered high risk according to platform benchmarks. For comparative analysis, we included paraquat (risk coefficient 0.982) as a known respiratory toxicant.

### Potential target screening

Protein targets potentially interacting with Quinacrine acetate were identified through searches in ChEMBL (Nowotka et al., 2017) (https://www.ebi.ac.uk/chembl/) and STITCH (http://stitch.embl.de/) databases using “Quinacrine acetate” as the keyword. We integrated and deduplicated the ChEMBL numbers and STITCH codes of potential targets, and standardized the nomenclature of obtained targets using the UniProt database to establish a Quinacrine acetate target library. Based on toxicity prediction results, we identified relevant targets through comprehensive searches of domestic and international literature, GeneCards, and OMIM databases using “respiratory system” and “respiratory system diseases” as keywords. A Venn diagram was constructed to determine the intersection between the Quinacrine acetate target library and the respiratory system toxicity target library, which served as the potential targets for this study.

### Functional enrichment and pathway analysis

GO analysis was performed using R (version 4.4.1) packages “clusterProfiler,” “enrichplot,” and “ComplexHeatmap” to elucidate the main biological functions of the targets in terms of biological processes (BP), cellular components (CC), and molecular functions (MF). KEGG pathway enrichment analysis of potential targets was conducted using “ggpubr” and “RColorBrewer” packages to identify key signaling pathways potentially affected by Quinacrine acetate. A threshold of FDR < 0.05 was set for significance to screen primary toxicity pathways.

### Protein-protein interaction network construction and core target screening

Potential targets were imported into the STRING database with a protein interaction confidence threshold >0.4 to construct a protein-protein interaction network. Cytoscape software was used for network topology analysis, and core hub genes were screened based on parameters including node degree, betweenness centrality, and closeness centrality. For the STRING database analysis, we selected a medium confidence threshold of >0.4 to capture a broader range of potential interactions while maintaining reasonable specificity. While Szklarczyk et al. (2019) recommend >0.7 for high-confidence networks, our exploratory toxicity analysis benefits from including more potential interactions to avoid missing relevant targets.

### Molecular docking analysis

Core target proteins were selected and their structures were obtained from the RCSB PDB database (https://www.rcsb.org/). Molecular docking was employed to further analyze the intermolecular interactions between Qac and the core target proteins identified in the study through predictions of binding modes and affinities. Specifically, CB-Dock2 database (Liu et al., 2022; Yang et al., 2022) (https://cadd.labshare.cn/cb-dock2/php/index.php) was used for molecular docking to evaluate the binding capability of Quinacrine acetate with core targets, further validating the network analysis results.

### Disease model validation

Based on the screened core targets, GSE5058 and GSE8545 datasets, comprising 60 normal patients and 33 COPD patients, were downloaded from the GEO database (https://www.ncbi.nlm.nih.gov/geo/) to analyze the expression patterns of these genes in COPD patients as a respiratory system disease example, thereby validating the correlation between targets and disease. GSE5058 and GSE8545 datasets were selected based on their comprehensive clinical annotation, platform compatibility, and sample quality assessment scores. Power analysis indicated that the combined sample size (60 normal patients and 33 COPD patients) provides 85% power to detect differentially expressed genes with a fold change ≥1.5 at a significance level of 0.05, which is sufficient for our exploratory analysis. Among them, GSE5058 includes 9 females, 30 males, 12 non-smokers and 27 smokers. GSE8545 includes 13 females, 41 males, 18 non-smokers and 27 smokers. Both datasets used the Affymetrix Human Genome U133 Plus 2.0 gene chip platform for gene expression analysis, and sample collection and processing methods followed standard operating procedures. These two datasets were selected because they collectively cover COPD patients of different severity, enhancing the universality and representativeness of the research results. These datasets were specifically chosen over other available COPD datasets because they include bronchial epithelial cell samples, which are most relevant to respiratory toxicity assessment. ROC curves based on core targets were constructed to evaluate the potential value of these genes as diagnostic markers for COPD. Additionally, the correlation between core targets and immune cell infiltration was analyzed to elucidate the immune regulatory role in Qac toxicity mechanisms. We have performed cross-validation using an independent dataset (GSE20257) to validate our model’s performance. The DSigDB database (https://dsigdb.tanlab.org/DSigDBv1.0/) was used to screen drugs related to core targets and predict possible drug-target-disease associations, providing insights for toxicity intervention.

## Results

### Toxicity prediction of quinacrine acetate

ProTox and ADMETlab analysis results revealed that Qac exhibited significant toxicity risk to the respiratory system, with a toxicity level of 4 (moderate toxicity) and a risk coefficient of 0.959, suggesting potential adverse effects on the respiratory system. The respiratory toxicity of Qac and paraquat is shown in Supplementary Table S2.

### Potential target screening and protein-protein interaction network

**FIGURE 1:**
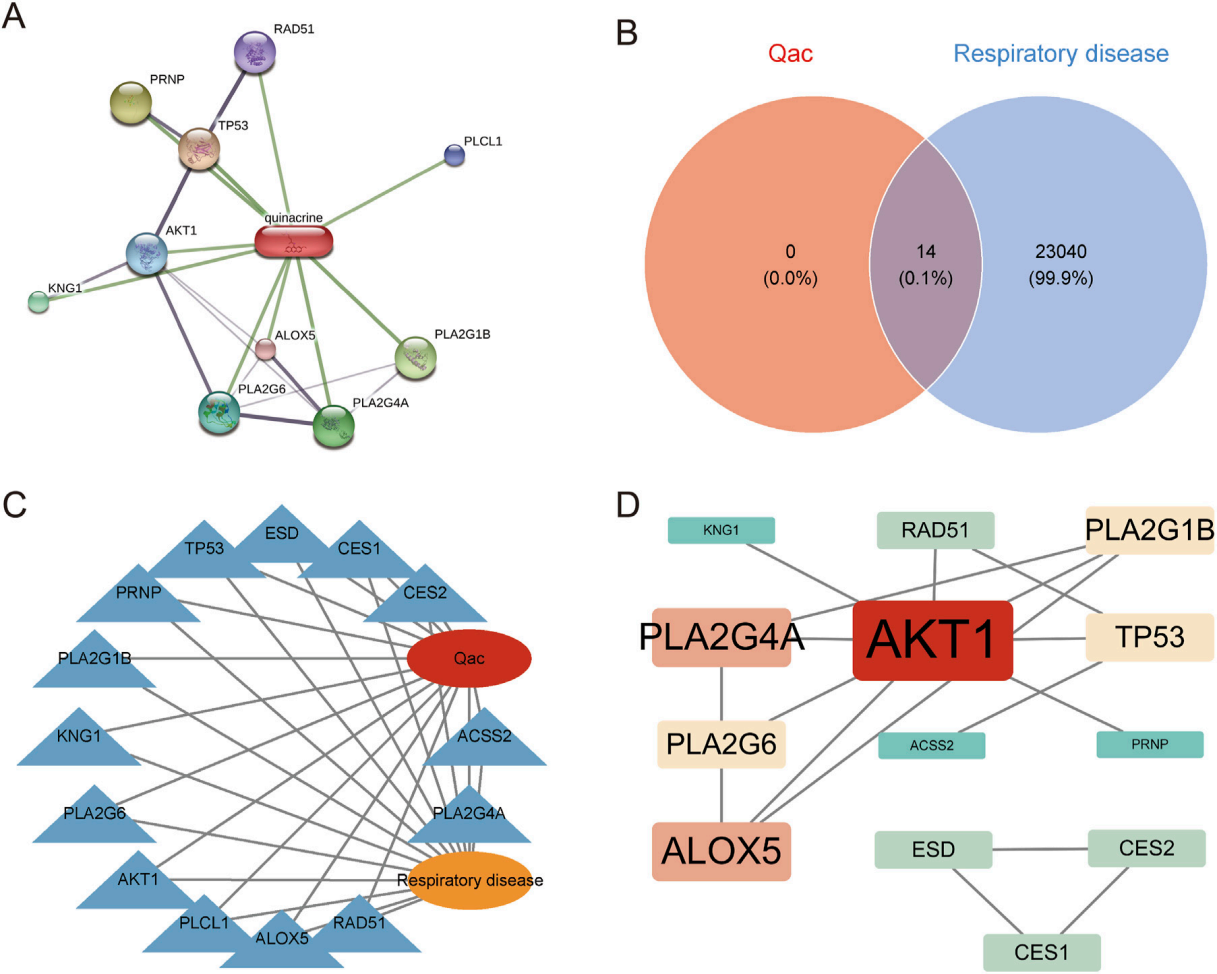
Network analysis of Quinacrine acetate and respiratory disease-related targets. **(A)** Protein-protein interaction network of Quinacrine acetate targets. The red node represents Quinacrine, and green lines indicate interactions with various targets. Protein nodes are displayed in different colors, with node size reflecting interaction intensity. **(B)** Venn diagram analysis of Quinacrine acetate (Qac) and respiratory system disease targets. **(C)** Network diagram depicting the relationship between Quinacrine acetate and respiratory system diseases. The central red node represents Qac, and the orange node represents respiratory system diseases. **(D)** Protein-protein interaction network of core targets. The red node represents AKT1, while red-orange nodes represent key targets such as PLA2G4A and ALOX5. Node size is proportional to degree centrality values, and lines indicate protein-protein interaction relationships.

Based on ChEMBL and STITCH, we identified 14 target genes related to Qac (Figure 1A). Cross-analysis with respiratory system-related disease genes showed an intersection of 14 common targets as illustrated in the Venn diagram (Figure 1B), which were determined to be potential targets for Qac-induced respiratory system toxicity. The network consists of 16 nodes (14 gene targets, 1 compound node for Qac, and 1 disease node) and 28 edges. The network exhibits an average path length of 1.87 and a modularity index of 0.42, indicating a moderately structured network with efficient information flow (Figure 1C). These metrics support our identification of key interaction patterns between Quinacrine acetate and respiratory disease-related targets. The protein-protein interaction network constructed based on the STRING database (Figure 1D) showed that Qac interacted with multiple targets, including AKT1, TP53, PRNP, and PLCL1. Through network topology analysis, three core hub genes were identified: AKT1, PLA2G4A, and ALOX5. These core targets displayed high degrees of connectivity and centrality in the network, indicating their potential critical roles in Qac-induced toxic responses.

### Functional enrichment and pathway analysis

**FIGURE 2:**
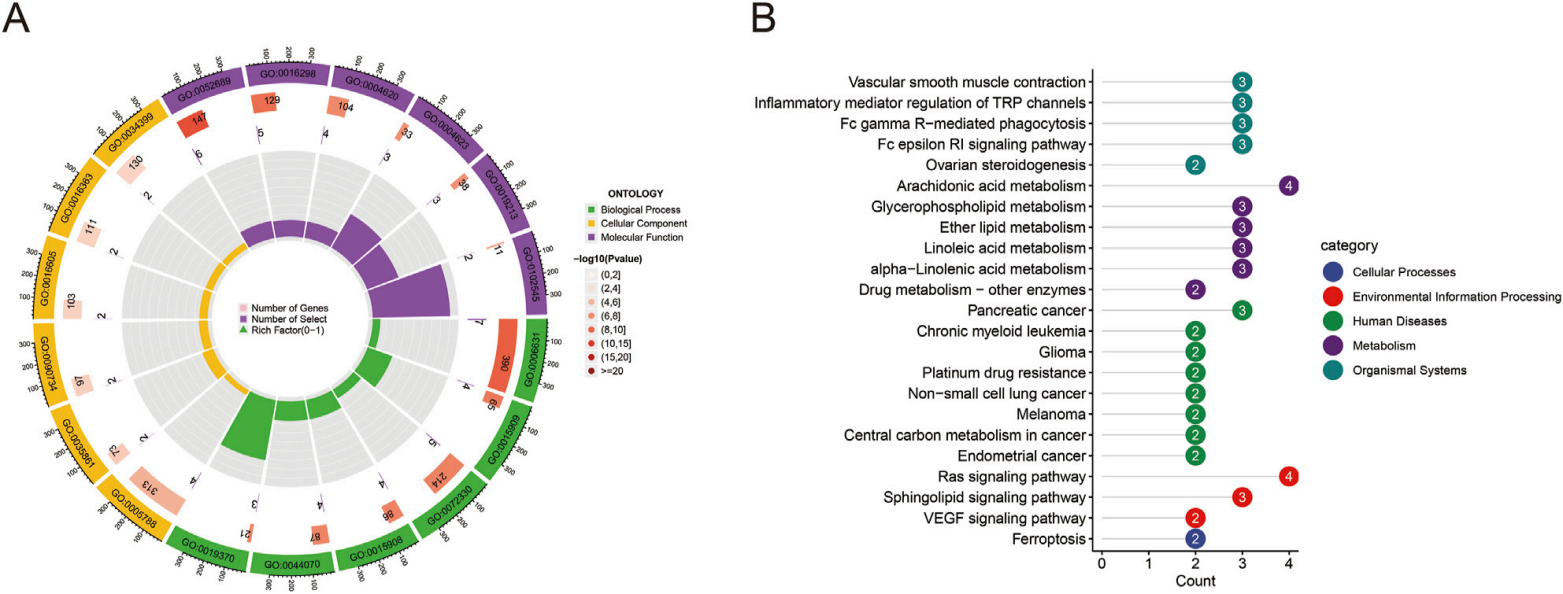
GO and KEGG enrichment analysis results. **(A)** Circular GO functional enrichment analysis of potential targets. The outer ring displays GO term categories (biological processes, cellular components, molecular functions), while the inner ring shows gene counts and statistical significance. Colors differentiate various GO functional categories. **(B)** KEGG pathway enrichment analysis of targets. The bar chart displays enriched pathways and their gene counts, with colors representing pathway classifications (cellular processes, environmental information processing, human diseases, metabolism, organismal systems). Numbers indicate the count of enriched genes in each pathway.

GO functional enrichment analysis (Figure 2A) indicated that the potential targets primarily participated in biological processes such as signal transduction, cell apoptosis, and lipid metabolism. KEGG pathway analysis (Figure 2B) demonstrated that these targets were mainly enriched in pathways including vascular smooth muscle contraction, inflammatory mediator regulation of TRP channels, Fc gamma R-mediated phagocytosis, and Fc epsilon RI signaling pathway. Notably, arachidonic acid metabolism, glycerophospholipid metabolism, and lipid metabolism pathways were significantly enriched, suggesting that Quinacrine acetate might induce respiratory system toxicity by affecting these pathways.

### Molecular docking analysis

**FIGURE 3:**
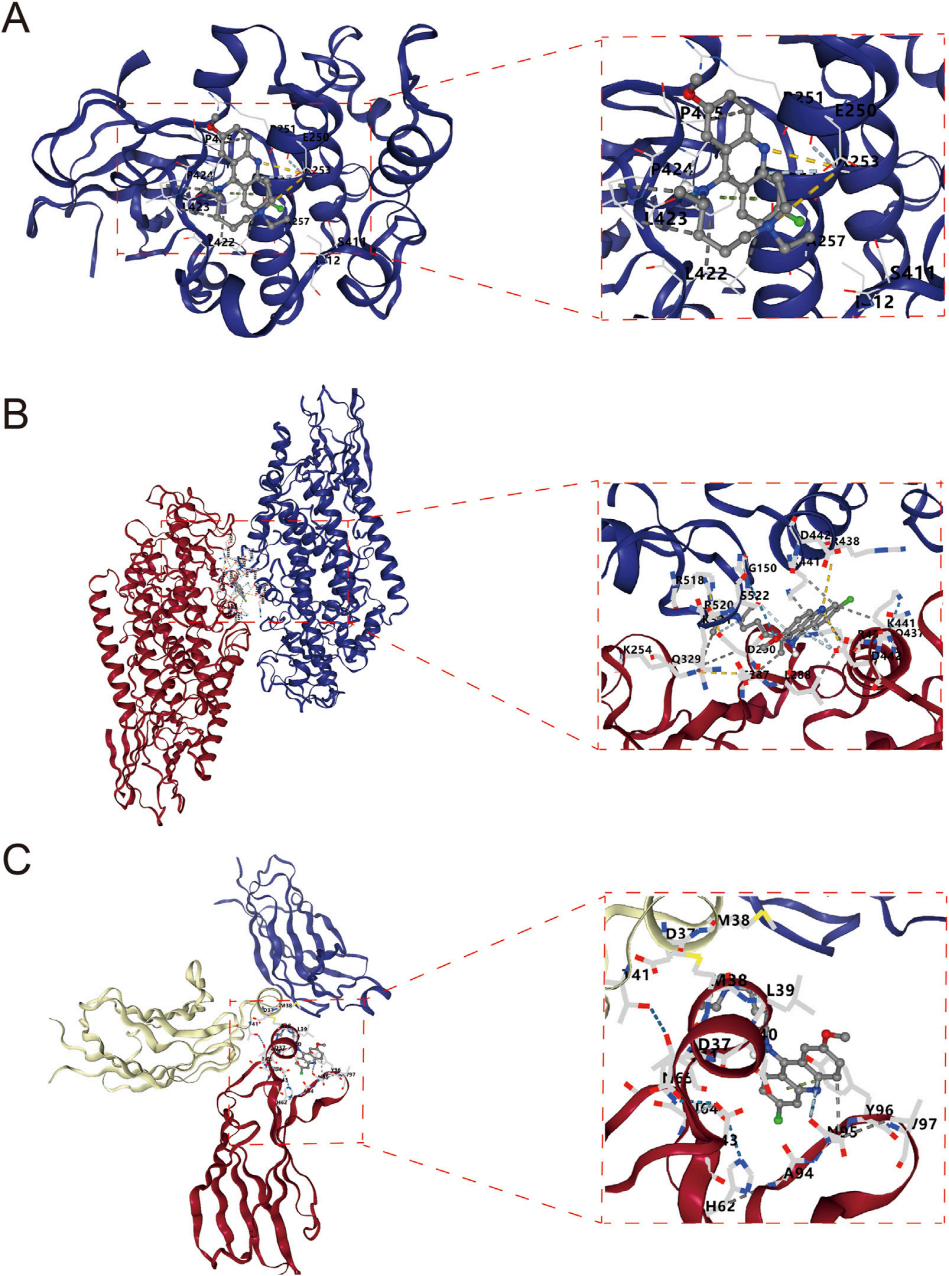
Molecular docking results of Quinacrine acetate with core targets. **(A)** Molecular docking model of Quinacrine acetate binding to AKT1 protein. The central red frame shows the 3D molecular structure of Quinacrine acetate. **(B)** Molecular docking results of Quinacrine acetate with PLA2G4A. **(C)** Molecular docking model of Quinacrine acetate with the ALOX5 target.

Figure 3 demonstrates the molecular docking results of quinacrine acetate with core targets. For AKT1 target (Figure 3A), we selected the C3 binding pocket (Vina score: −5.9, cavity volume: 580 Å^3^) as the optimal binding site, where quinacrine acetate forms stable interactions near position (−31, 117, −74). The relatively high Vina score indicates moderate binding affinity, with the compound primarily forming hydrophobic interactions through its quinoline ring while establishing hydrogen bonds with nearby residues (Table 1). For ALOX5 (Figure 3B), the C1 pocket showed the strongest binding (Vina score: −7.4, cavity volume: 1,915 Å^3^) near position (2, 46, 3), suggesting high binding affinity. This extensive binding pocket allows quinacrine acetate to establish multiple contact points near the catalytic center of ALOX5, potentially interfering with its enzymatic activity (Table 2). For PLA2G4A (Figure 3C), quinacrine acetate preferentially binds to the C1 pocket (Vina score: −6.1, cavity volume: 458 Å^3^) near position (−40, −8, 22). The molecular interactions in this region suggest that quinacrine acetate could affect the catalytic function of PLA2G4A by potentially blocking substrate access or altering protein conformation (Table 3). These molecular docking results provide structural evidence supporting our network analysis findings and suggest direct molecular mechanisms for quinacrine acetate’s effects on these core targets.

**TABLE 1:** Docking table of AKT1 and Quinacrine acetate.

CurPocket ID	Vina score	Cavity volume (Å3)	Center (x, y, z)	Docking size (x, y, z)
C1	−5.7	4,185	−29, 94, −59	32, 23, 23
C2	−5.7	782	−18, 97, −73	23, 23, 29
C3	−5.9	580	−31, 117, −74	23, 23, 23
C4	−5.2	463	−32, 93, −76	23,23, 23
C5	−4.4	149	−8, 108, −82	23, 23, 23

**TABLE 2:** Docking table of ALOX5 and Quinacrine-acetate.

CurPocket ID	Vina score	Cavity volume (Å3)	Center (x, y, z)	Docking size (x, y, z)
C1	−7.4	1,915	2, 46, 3	23, 23, 33
C2	−7.2	1,950	14, 57, 0	23, 23, 23
C3	−7.2	1,938	11, 38, 13	23, 23, 23
C4	−6.6	3,516	−1l, 77, 31	23, 23, 35
C5	−6.5	2,859	16, 15, −23	23, 23, 33

**TABLE 3:** Docking table of PLA2G4A and Quinacrine-acetate.

CurPocket ID	Vina score	Cavity volume (Å3)	Center (x, y, z)	Docking size (x, y, z)
C1	−6.1	458	−40, −8, 22	23, 23, 23
C2	−5.8	231	−82, −39, 10	23, 23, 23
C3	−5.8	193	−35, −43, 33	23, 23, 23
C4	−5.7	198	−31, −14, 23	23,23, 23
C5	−5.7	186	−7, 5, 12	23, 23, 23

### COPD disease model validation

**FIGURE 4:**
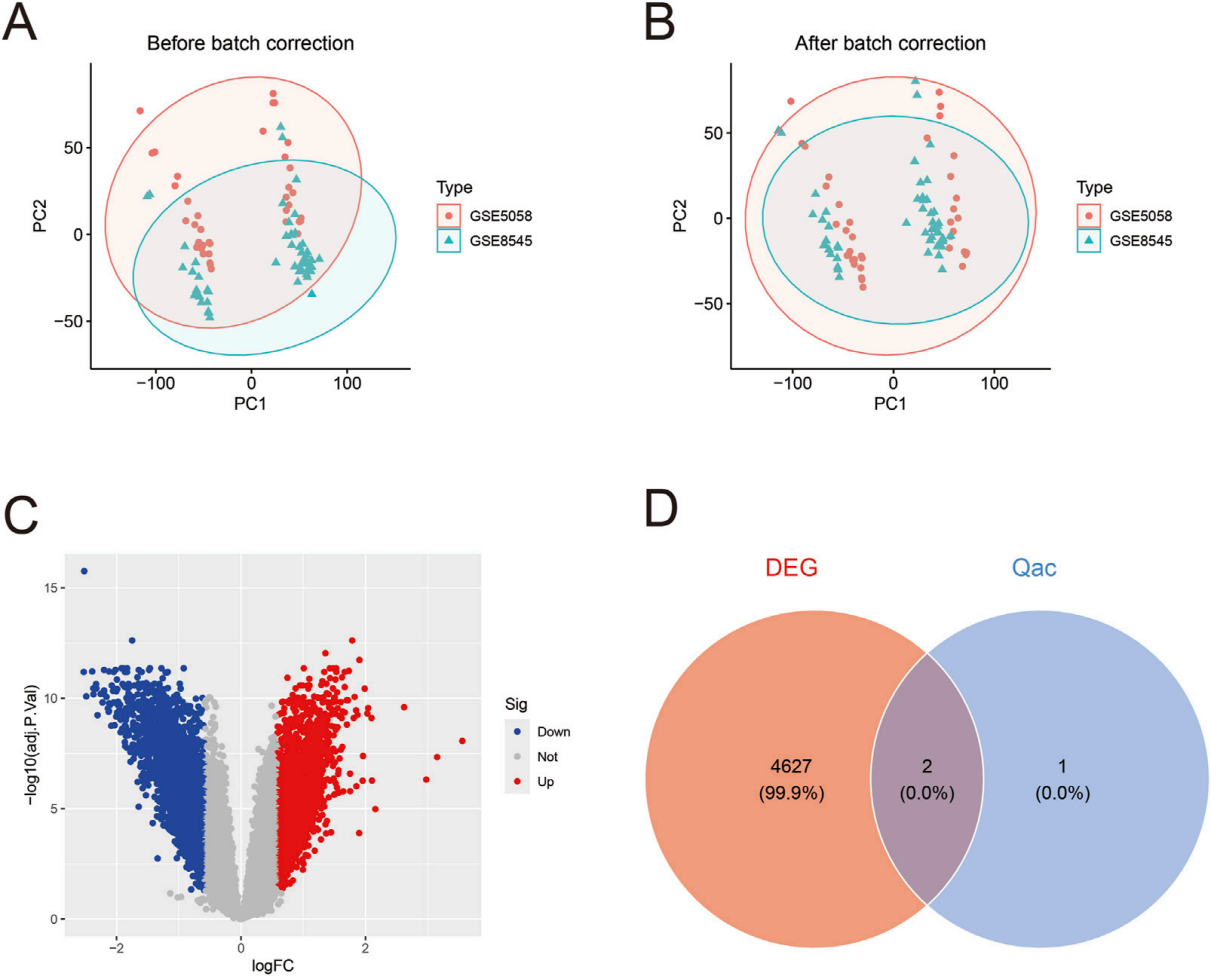
Gene expression data analysis of COPD patients. **(A)** Principal component analysis (PCA) before batch correction. Red dots and green triangles represent samples from GSE5058 and GSE8545 datasets respectively, with ellipses indicating 95% confidence intervals, showing evident batch effects. **(B)** PCA plot after batch correction. The distribution of samples from both datasets appears more uniform after correction, indicating effective elimination of batch effects and improved reliability for subsequent analyses. **(C)** Volcano plot of differentially expressed genes. Red dots represent upregulated genes, blue dots represent downregulated genes, and gray dots indicate genes without significant differences. **(D)** Venn diagram of differentially expressed genes (DEG) and Qac targets.

**FIGURE 5:**
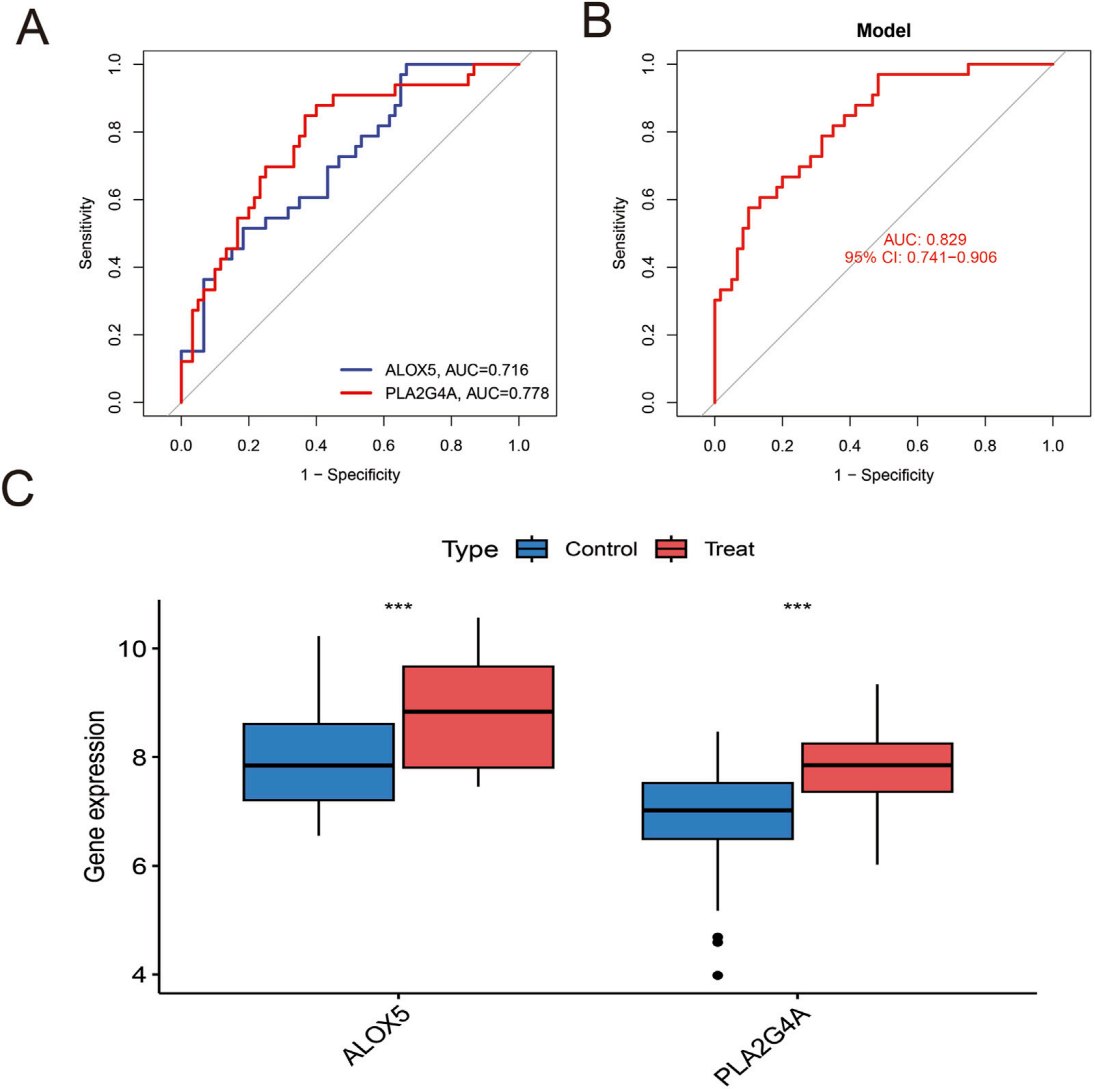
Analysis of Hub Gene Expression and Diagnostic Value. **(A)** ROC curve analysis of individual Hub genes. The blue line represents the ROC curve of ALOX5 (AUC = 0.716), and the red line represents the ROC curve of PLA2G4A (AUC = 0.778). **(B)** ROC curve of the predictive model based on the combination of Hub genes. The red line represents the model's ROC curve, with an AUC value of 0.829 (95% CI: 0.741–0.906), indicating that the combined model has excellent diagnostic value. **(C)** Comparison of Hub gene expression levels between the control group and the COPD group. Blue boxes represent the control group, and red boxes represent the COPD group. ALOX5 and PLA2G4A are significantly upregulated in COPD patients.

After batch effect correction of GEO datasets (Figures 4A,B), differential expression analysis revealed 2,305 upregulated genes and 2,305 downregulated genes (Figure 4C; Supplementary Table S1). Venn diagram cross-analysis showed that PLA2G4A and ALOX5 exhibited significant differences in COPD patients (Figure 4D). In COPD patients, PLA2G4A and ALOX5 were primarily located on chromosomes 1 and 10 (Supplementary Figure S1). ROC curve analysis (Figures 5A,B) indicated that the model based on core targets PLA2G4A and ALOX5 demonstrated good diagnostic performance, with AUC values of 0.778 and 0.716 respectively, while the combined model achieved an AUC of 0.829 (95% CI: 0.741–0.906), and both PLA2G4A and ALOX5 were significantly upregulated (Figure 5C). We have performed cross-validation using an independent dataset (GSE20257) to validate our model’s performance. As shown in the additional data provided, the ROC analysis confirms the robustness of our findings with AUC values of 0.711 (0.600–0.811) for ALOX5, 0.815 (0.720–0.902) for PLA2G4A, and 0.788 (0.694–0.872) for the combined model (Supplementary Figure S2A). These results support the generalizability of our findings across different patient populations.

### Core gene immune cell and drug target association analysis

**FIGURE 6:**
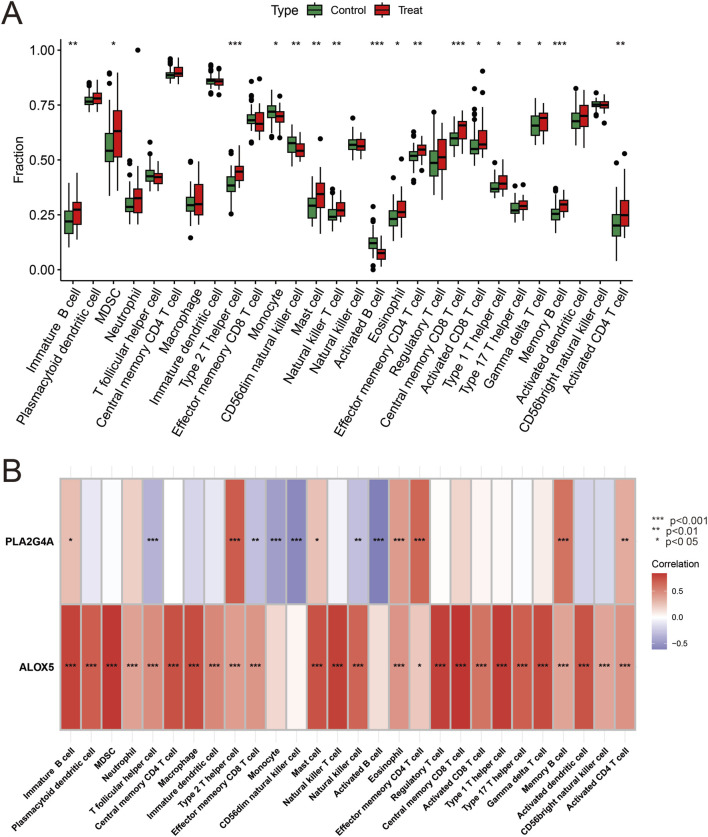
Hub Gene Expression and Immune Correlation. **(A)** Analysis of immune cell infiltration differences between the control group and the COPD group. Green boxes represent the control group, and red boxes represent the COPD group. **(B)** Heatmap analysis of the correlation between Hub genes and immune cells. ALOX5 and PLA2G4A show significant positive correlations with multiple immune cells. Red indicates positive correlation, blue indicates negative correlation, and the color intensity represents the strength of the correlation.*P<0.05, **P < 0.01, ***P < 0.001.

**FIGURE 7:**
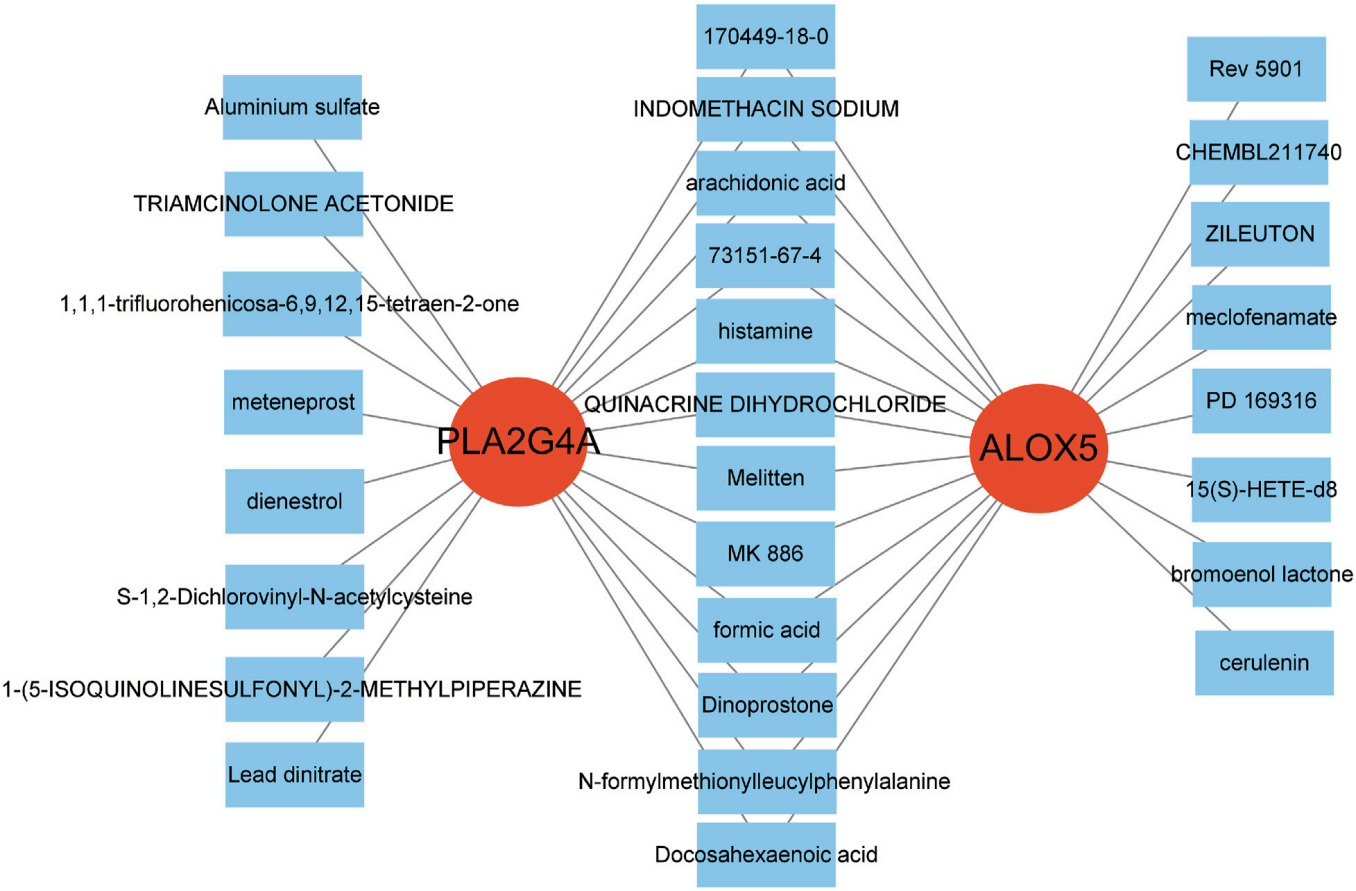
Drug-Target Network Associated with Hub Genes. The two red central nodes represent the core targets, PLA2G4A and ALOX5. The surrounding blue squares denote drugs or compounds associated with these targets.

Analysis of immune cell expression in COPD patients using “GSVA” and “GSEABase” showed that most immune cells were highly expressed in COPD patients (Figure 6A). We have revised our analysis to include FDR control using the Benjamini-Hochberg procedure. Immune infiltration analysisrevealed that ALOX5 and PLA2G4A were positively correlated with various immune cell expressions, suggesting that Qac might participate in the development of respiratory system diseases by regulating immune responses through these targets, now displays only correlations that remain significant after FDR correction (Figure 6B). In addition, the validation set GSE20257 demonstrates the expression of multiple immune cells in COPD and their association with PLA2G4A and ALOX5 (Supplementary Figure S2B,C). Drug target network analysis (Figure 7) demonstrated that core targets ALOX5 and PLA2G4A served as targets for various drugs. PLA2G4A (cytosolic phospholipase A2) is shown to interact with 20 distinct compounds, including quinacrine dihydrochloride, which is structurally related to our compound of interest. Among these interactions, several compounds exhibit dual-targeting properties, simultaneously affecting both PLA2G4A and ALOX5. These include quinacrine dihydrochloride, histamine, Melitten, arachidonic acid, formic acid, MK 886, N-formylmethionylleucylphenylalanine, Dinoprostone, and compound 73151-67-4. This dual-targeting pattern suggests a potential mechanism through which quinacrine acetate might amplify its effects on the arachidonic acid pathway by simultaneously modulating multiple enzymes. The network also reveals that ALOX5 interacts with 19 compounds, including several specific inhibitors and substrates that modulate the production of leukotrienes. The interaction with bronoenol lactone and cerulenin suggests potential modulation of enzyme activity through lipid metabolism pathways beyond direct catalytic inhibition.

## Discussion

Network toxicology, as an emerging research strategy, provides a unique perspective for systematically understanding the toxicity mechanisms of small molecule compounds. This study applies this strategy to reveal the potential toxicity mechanisms of quinacrine acetate as a cGAS-STING-TBK1 pathway modulator and validates these findings through molecular-level and disease model validations. Compared to traditional toxicological methods, our network toxicology strategy can more comprehensively capture drug-target-disease relationships, with significant advantages in revealing off-target effects. Our research is the first to elucidate from a systems biology perspective the mechanism by which quinacrine acetate may increase respiratory system disease risk through multi-target actions, providing new scientific basis for the safe application of this compound.

In our study, we identified AKT1 as a core target that plays a crucial role in various biological processes. AKT1 is a core component of the PI3K/AKT signaling pathway, which is important in cell proliferation, survival, and metabolism (Manning and Toker, 2017). Recent studies have shown that abnormal activation of AKT1 is closely associated with various respiratory system diseases, including COPD (Liang et al., 2023), pulmonary fibrosis (Yan et al., 2024), and lung cancer (Tian et al., 2022). Our molecular docking results demonstrate that Quinacrine acetate can form stable bonds with AKT1, with low binding energy, suggesting that it might interfere with downstream signal transduction by inhibiting AKT1 activity. Hao et al. (2020) confirmed that AKT1 inhibition increases apoptosis, which may be an important mechanism underlying Quinacrine acetate-induced respiratory system toxicity.

PLA2G4A (cytosolic phospholipase A2) serves as a key enzyme in arachidonic acid metabolism and plays a central role in initiating and maintaining inflammatory responses (Murakami et al., 2011). Jiang et al. (2023) research found that upregulation of PLA2G4A positively correlates with the severity of airway inflammation in COPD patients. Our results suggest that Quinacrine acetate may alter arachidonic acid metabolic balance by affecting PLA2G4A activity, thereby leading to dysregulated inflammatory responses.

ALOX5 (5-lipoxygenase) is another core target identified in our study that catalyzes the conversion of arachidonic acid to leukotrienes, participating in inflammatory and allergic reactions (Werz et al., 2018). Nunns et al. (2018) research indicated that abnormal expression of ALOX5 is associated with lung injury. The combination model based on PLA2G4A and ALOX5 achieved an AUC value of 0.829, indicating the potential value of these two markers in COPD diagnosis. Currently, COPD diagnosis mainly relies on pulmonary function tests and imaging examinations, lacking specific biomarkers. Our findings provide a new candidate marker combination for molecular diagnosis of COPD, with particular potential value for screening early COPD patients. This diagnostic model may achieve non-invasive diagnosis through detection of PLA2G4A and ALOX5 expression levels in peripheral blood, reducing the burden on patients. Additionally, these two markers may reflect the inflammatory state and disease progression of COPD, with potential significance for guiding individualized treatment decisions. Future research could further validate the sensitivity and specificity of this diagnostic model in larger prospective cohorts and explore its application value in monitoring treatment effects and predicting disease prognosis.

The positive correlation between ALOX5 and PLA2G4A with various immune cells has important biological significance. PLA2G4A, as cytosolic phospholipase A2, plays a key role in the release of arachidonic acid, while ALOX5 participates in the conversion of arachidonic acid to leukotrienes. The coordinated upregulation of these two enzymes may lead to excessive production of pro-inflammatory leukotriene products (such as LTB4 and cysteinyl leukotrienes), which are potent neutrophil and eosinophil chemoattractants. Our immune cell correlation analysis shows that ALOX5 and PLA2G4A have the strongest positive correlation with neutrophils and CD8^+^ T cells, consistent with the immunopathological characteristics of COPD, namely, the core role of neutrophils and CD8^+^ T cells in airway inflammation and tissue remodeling (Peters-Golden and Henderson, 2007). This correlation suggests a possible mechanism: quinacrine acetate affects ALOX5 and PLA2G4A activity, alters leukotriene metabolic balance, promotes recruitment and activation of specific immune cells, ultimately leading to persistent inflammatory responses and tissue damage. Additionally, the correlation between ALOX5 and PLA2G4A with macrophages suggests the potential role of these enzymes in macrophage polarization and functional transformation, which may influence the pathogenesis and progression of respiratory system diseases.

Abnormalities in cellular signal transduction pathways form the molecular basis for various diseases. Our results indicate that Quinacrine acetate may affect vascular smooth muscle contraction, inflammatory mediator regulation of TRP channels, and Fc receptor signaling pathways. These pathways play important roles in the normal physiological function and pathological states of the respiratory system (Talbot et al., 2015). Shang et al. (2016) research confirmed that small molecule compounds interfering with vascular smooth muscle contraction pathways can lead to pulmonary vascular dysfunction and respiratory system damage. Quinacrine acetate may increase the risk of respiratory system diseases by affecting these pathways, resulting in abnormal pulmonary vascular and airway function.

Zhang et al. (2022) employed a similar network toxicology approach in studying the toxicity of citric acid ester plasticizers, but they primarily focused on digestive system toxicity, while our research focuses on the respiratory system, expanding the application of network toxicology in system-specific toxicity assessment. Compared to Hao et al.’s research on AKT1 inhibition leading to apoptosis, our findings further reveal the key role of AKT1 in respiratory system toxicity and provide evidence of direct interaction between quinacrine acetate and AKT1 through molecular docking. Jiang et al. (2023) found that PLA2G4A upregulation positively correlates with airway inflammation severity in COPD patients, and our research not only confirms this observation but also elucidates the mechanism by which quinacrine acetate may alter arachidonic acid metabolic balance by affecting PLA2G4A activity through network analysis and molecular docking, establishing a potential causal link between the two. Shang et al. (2016) confirmed that small molecule compounds interfering with vascular smooth muscle contraction pathways can lead to pulmonary vascular dysfunction, and our research, through KEGG pathway analysis, confirms that quinacrine acetate indeed affects this pathway, further supporting its potential mechanism in causing respiratory system damage.

Immune regulation plays a key role in the development of respiratory system diseases. Our immune cell association analysis shows that the core targets ALOX5 and PLA2G4A are closely associated with the expression of various immune cells. Quinacrine acetate may participate in the pathological process of respiratory system diseases by affecting the activity of ALOX5 and PLA2G4A, thereby altering immune cell function and distribution.

The cGAS-STING-TBK1 signaling pathway is an important innate immune pathway that plays a key role in recognizing cytoplasmic DNA and activating inflammatory responses (Chen et al., 2016). In recent years, abnormal activation of this pathway has been associated with various autoimmune and inflammatory diseases, including systemic lupus erythematosus, STING-associated vasculopathy, and pulmonary fibrosis (Haag et al., 2018). Li et al. (2016) research showed that excessive activation of the cGAS-STING-TBK1 signaling pathway can lead to lung inflammation and fibrosis, while targeted inhibition of this pathway can alleviate lung injury. As a small molecule modulator of the cGAS-STING-TBK1 signaling pathway, the therapeutic potential and toxicity risks of Quinacrine acetate have received significant attention. Our study is the first to reveal from a network toxicology perspective that Quinacrine acetate may increase respiratory system toxicity risk through multi-target actions, especially by affecting AKT1, PLA2G4A, and ALOX5, interfering with arachidonic acid metabolism and inflammatory responses. This discovery provides a new perspective for understanding the therapeutic effects and toxic effects of Quinacrine acetate.

This study represents a significant advance in applying network toxicology strategies to elucidate the toxicity mechanisms of small molecule modulators. Our findings demonstrate that quinacrine acetate may increase respiratory system disease risk by targeting AKT1, PLA2G4A, and ALOX5, affecting metabolic pathways and inflammatory responses. Recent breakthroughs by Haag et al. (2018) on allosteric inhibition mechanisms of STING and Li et al. (2023) on cGAS-STING’s connection to metabolic reprogramming provide context for our findings, suggesting potential crosstalk between the cGAS-STING-TBK1 pathway and the metabolic pathways we identified. The network toxicology approach demonstrated here offers distinct advantages over traditional toxicology methods, including higher throughput, mechanistic insights, and the ability to predict off-target effects before costly *in vivo* testing. For clinical applications, our findings suggest careful respiratory monitoring during quinacrine acetate treatment, particularly in patients with pre-existing respiratory conditions like COPD. Future therapeutic development could focus on structural modifications to reduce binding affinity to AKT1, PLA2G4A, and ALOX5 while maintaining cGAS-STING-TBK1 pathway modulation.

Traditional toxicological research primarily relies on *in vivo* and *in vitro* experiments, which are time-consuming, costly, and often fail to comprehensively reveal the molecular mechanisms of toxic effects (Hartung, 2009). In recent years, with the rapid development of bioinformatics and systems biology, network toxicology has emerged as a novel toxicological research strategy that provides new insights for comprehensively analyzing compound toxicity mechanisms by integrating multi-omics data and constructing molecular interaction networks (Tao et al., 2013). Huang (2023) research confirmed that network toxicology can effectively predict the toxic targets and mechanisms of action of environmental pollutants, providing a scientific basis for toxicity risk assessment.

This study is the first to apply network toxicology strategies to investigate the toxicity mechanisms of Quinacrine acetate, comprehensively analyzing its potential toxicity mechanisms in the respiratory system by integrating multiple databases and analytical tools. Compared to traditional methods, this strategy offers advantages in efficiency, comprehensiveness, and systematicity, enabling rapid prediction of compound toxic targets and action pathways to guide subsequent experimental validation. Meanwhile, the application of molecular docking technology further validates the interactions between small molecules and target proteins, enhancing the reliability of the research results.

An important limitation of this study is the lack of functional validation experiments on quinacrine acetate as a regulator of the cGAS-STING-TBK1 signaling pathway. Although network analysis and molecular docking results provide insights into the potential toxicity mechanisms of quinacrine acetate, future research should validate its direct regulatory effects on key components of the cGAS-STING-TBK1 pathway such as cGAS, STING, and TBK1 through *in vitro* and *in vivo* experiments, as well as the relationship between this regulation and the off-target effects we discovered. Specifically, the direct link between quinacrine acetate exposure and changes in AKT1, PLA2G4A, and ALOX5 expression should be established, and how these changes affect downstream inflammatory and immune responses should be explored. Additionally, constructing cell models with specific knockdown or overexpression of AKT1, PLA2G4A, and ALOX5 to observe their effects on quinacrine acetate-induced cytotoxicity is an important strategy to validate our prediction results.

To verify the network analysis and prediction results of this study, we propose the following specific verification strategies: (1) Using *in vitro* cell models, such as human bronchial epithelial cells and alveolar epithelial cells, to verify changes in expression and activity of AKT1, PLA2G4A, and ALOX5 after quinacrine acetate treatment through Western blot and qRT-PCR techniques; (2) Applying CRISPR-Cas9 gene editing technology to construct cell lines with AKT1, PLA2G4A, and ALOX5 knockout or mutation, observing the effects of gene modification on quinacrine acetate-induced cytotoxicity and inflammatory responses; (3) Determining the direct binding affinity between quinacrine acetate and predicted targets through surface plasmon resonance (SPR) or isothermal titration calorimetry (ITC) to validate molecular docking results; (4) Establishing a mouse model exposed to quinacrine acetate, verifying its toxic effects on the respiratory system and changes in related targets and pathways through tissue pathology, immunohistochemistry, and single-cell RNA sequencing technology; (5) Designing targeted intervention strategies based on predicted targets, such as using specific inhibitors or agonists, to evaluate their antagonistic or synergistic effects on quinacrine acetate toxicity. These verification strategies will comprehensively validate the prediction results of this study from molecular, cellular, and animal levels, providing a solid foundation for in-depth understanding of quinacrine acetate toxicity mechanisms.

## Conclusion

This study utilized network toxicology and molecular docking strategies to reveal the potential toxicity mechanisms of Quinacrine acetate as a small molecule modulator of the cGAS-STING-TBK1 signaling pathway. Our findings demonstrate that Quinacrine acetate may increase the risk of respiratory system diseases by targeting core genes including AKT1, PLA2G4A, and ALOX5, thereby affecting metabolic pathways and inflammatory responses. These discoveries provide a theoretical foundation for understanding the toxic effects of Quinacrine acetate and offer new perspectives and methodological references for evaluating the toxicity of small molecule compounds in respiratory system diseases.

## Data Availability

The datasets presented in this study can be found in online repositories. The names of the repository/repositories and accession number(s) can be found in the article/Supplementary Material.
