# Supplementary figures and images for: Toxicity mechanism analysis of cGAS-STING-TBK1 signaling pathway small molecule modulator based on network toxicology and molecular docking strategy: quinacrine acetate as an example
Source: Front Chem. 2025 Apr 22;13:1584588. doi: 10.3389/fchem.2025.1584588 (PMC12052562; doi:10.3389/fchem.2025.1584588)

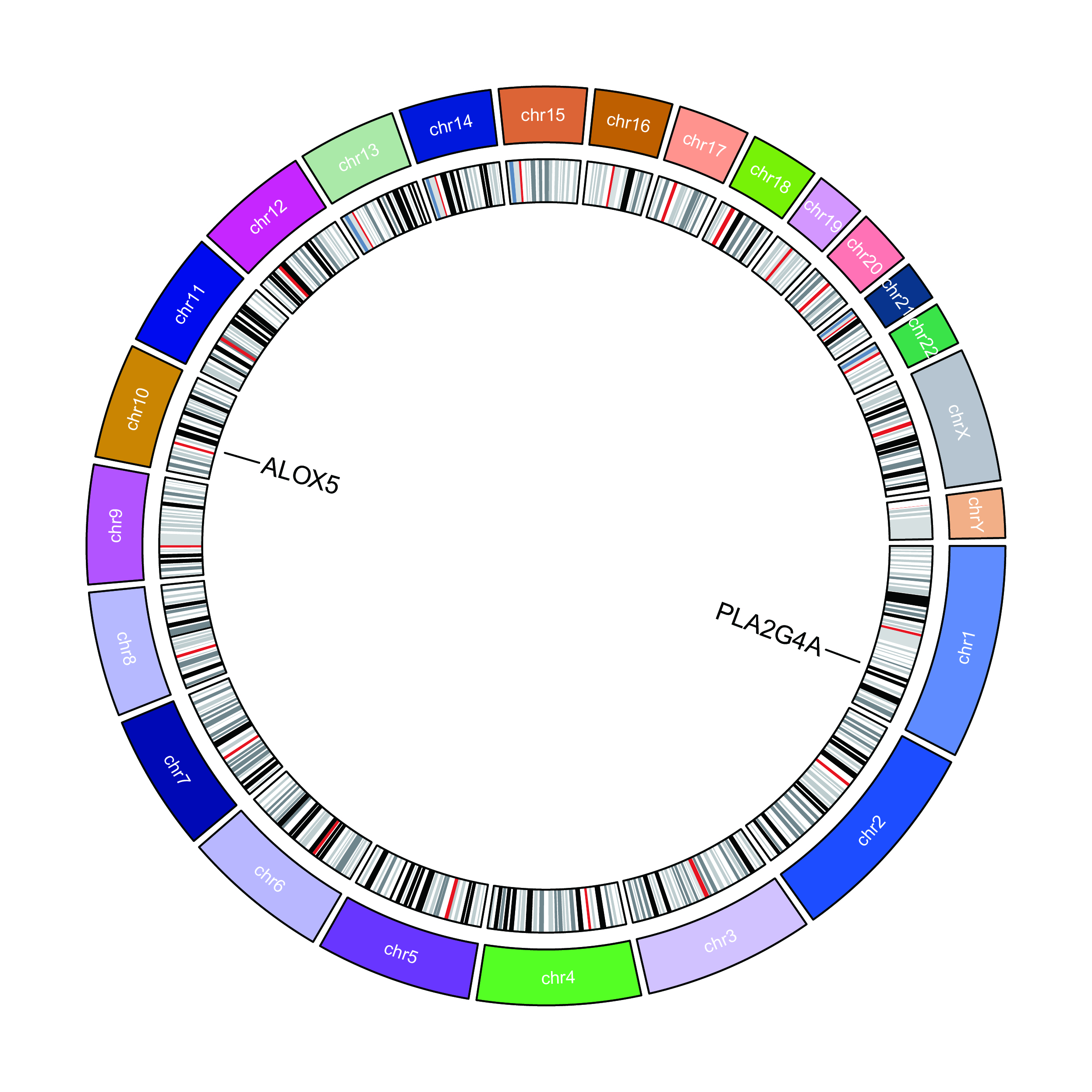

Supplement: Supplementary file 1 [file Image1.jpeg]

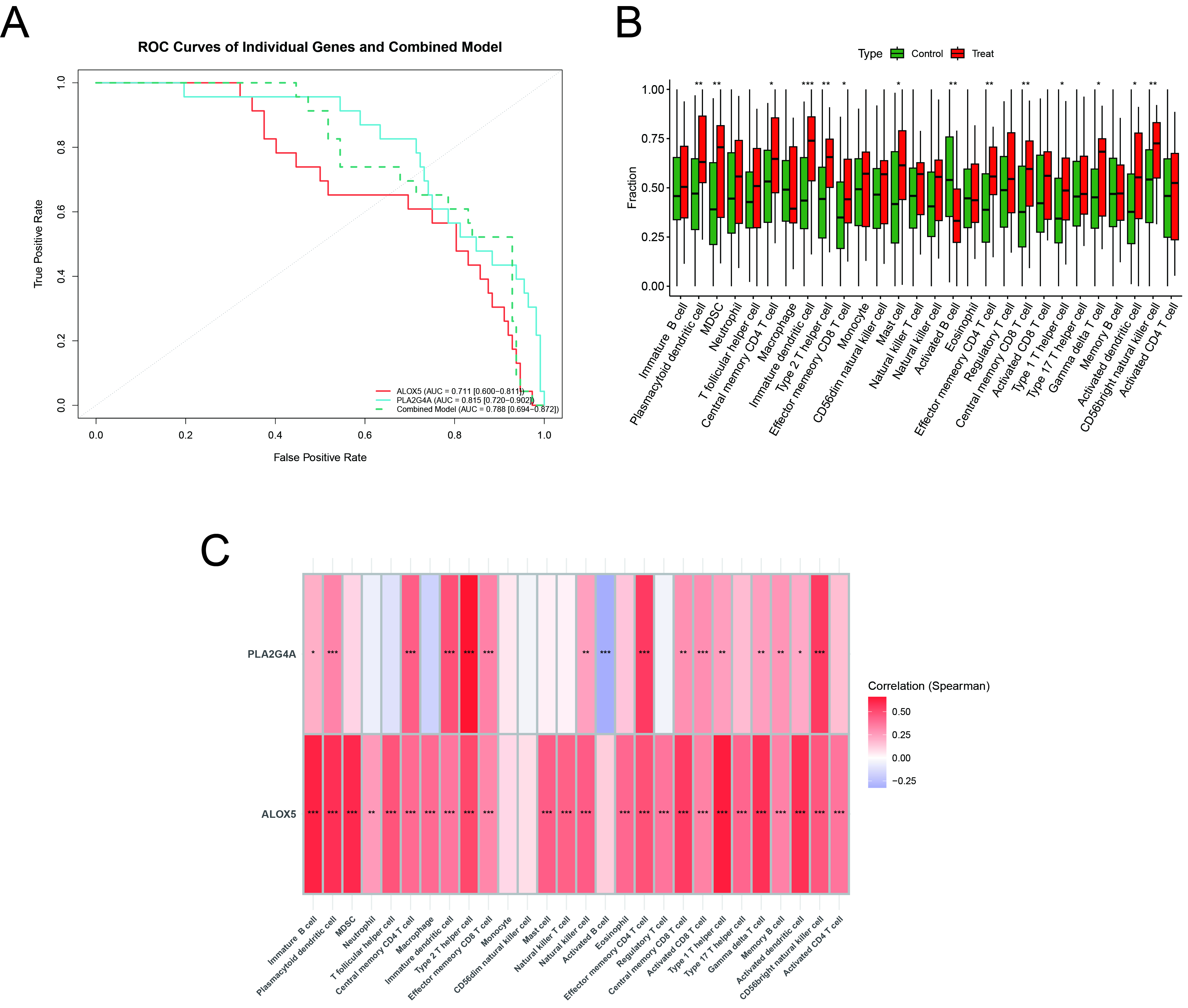

Supplement: Supplementary file 2 [file Image2.jpeg]
