# Supplementary material for: Toxicity mechanism analysis of cGAS-STING-TBK1 signaling pathway small molecule modulator based on network toxicology and molecular docking strategy: quinacrine acetate as an example
Source: Front Chem. 2025 Apr 22;13:1584588. doi: 10.3389/fchem.2025.1584588 (PMC12052562; doi:10.3389/fchem.2025.1584588)
Supplement: Supplementary file 3 [file Table2.docx]

| Toxicity of compounds | ProTox(prediction) | ADMETlab(**Value**) |
| --- | --- | --- |
| Quinacrine-acetate | Active, 0.98 | 0.959 |
| paraquat | Active, 0.87 | 0.87 |

Supplementary Table2

Category 1: respiratory toxicants; Category 0: respiratory nontoxicants. The output value is the probability of being toxic, within the range of 0 to 1.
